# Creeping Attachment Involving Dental Implants: Two Case Reports with a Two-Year Follow-Up from an Ongoing Clinical Study

**DOI:** 10.1155/2014/756908

**Published:** 2014-09-03

**Authors:** Armando R. Lopes Pereira Neto, Bernardo Born Passoni, José Moisés de Souza, João Gustavo Oliveira de Souza, César Augusto Magalhães Benfatti, Ricardo de Souza Magini, Marco Aurélio Bianchini

**Affiliations:** Federal University of Santa Catarina, 88040-900 Florianópolis, SC, Brazil

## Abstract

*Introduction*. This paper describes case reports where coronal growth of soft tissue on implant threads was observed after surgery for soft tissue graft. This phenomenon is known as “creeping attachment.” *Methods*. Two patients were submitted to gingival graft procedure including subepithelial connective tissue graft and masticatory mucosal graft. A two-year follow-up appointment was performed. *Results*. After a two-year follow-up gingival growth over titanium surfaces characterizing the “creeping attachment” phenomenon was observed. This gingival growth happened over abutment and threads surfaces. *Conclusion*. The creeping attachment phenomenon is possible over titanium surfaces and has not yet been reported in the relevant literature over this kind of structure.

## 1. Introduction

The use of soft tissue grafts in implantology has been widely explored in the literature [1, 2]. Increased width of keratinized mucosa, volume augmentation around the implant and pontic areas, and sealing of extraction sites are some of the indications for surgical procedures of this type [3–6]. Among the techniques used, the free gingival graft of keratinized tissue can be highlighted [1]. Some of these mentioned techniques are intended to improve the esthetics, while others seek to restore health to the peri-implant tissue [2].

In order to prevent the increase of recession, keratinized mucosal grafts described in the end of the 60s can be applied for teeth and around implants [7]. In this type of procedure, the goal is not intended to cover threads or exposed roots, although some authors had described a technique using donor material with thickness of 2 mm for this purpose [8]. This technique has the main goal to create a thick mucosa with superior quality, increasing the resistance to mechanical trauma from tooth brushing and enabling marginal homeostasis [9].

However, a coronal margin growth of soft tissue on the exposed roots in the postoperative recovery of soft tissue grafts was observed. This phenomenon was called “creeping attachment” [10]. Some authors observed this phenomenon especially in the anterior mandible region [11–14] and also a single case in the anterior maxilla [15]. Thus, this paper presents two clinical cases in which the “creeping attachment” was observed around implants where characteristics of the metal tend to preclude such phenomenon.

## 2. Case Reports

### 2.1. Case 1

**Figure 1:**
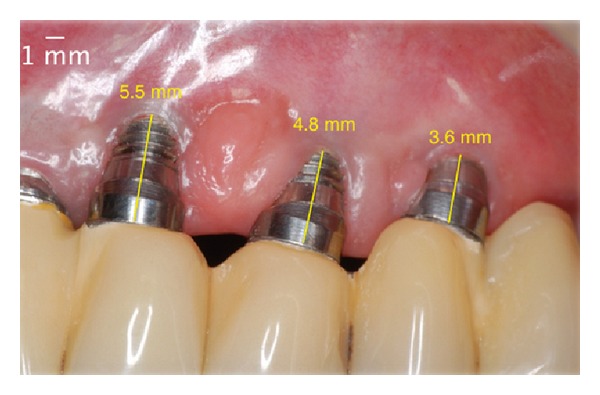
Initial appearance. Note exposure of threads and the absence of keratinized mucosa.

**Figure 2:**
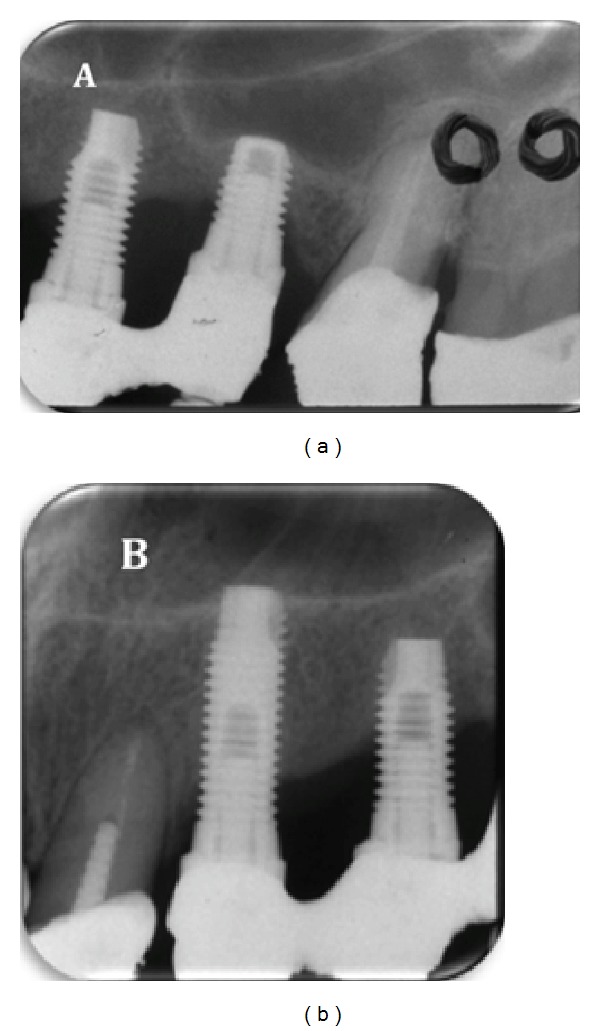
Radiographic initial appearance.

**Figure 3:**
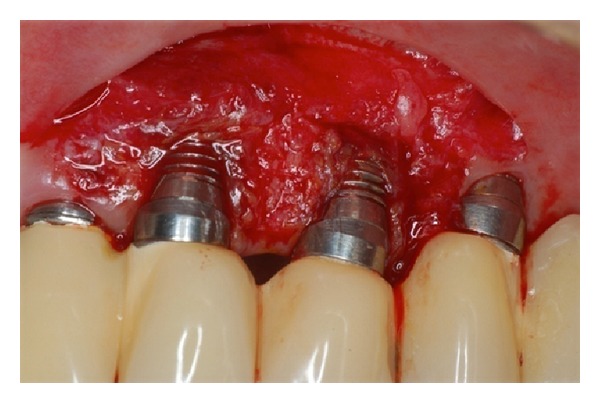
Recipient site prepared to receive the free gingival graft.

During a clinical examination, a female presented with recession of the buccal mucosa of implants located in the region teeth numbers 22, 23 and 24. Also, the lack of keratinized tissue in this region and some degree of inflammation of the soft tissues were observed that combined with radiographic images featured a diagnosis of peri-implantitis (Figures 1, 2, and 3). In order to stop the retraction of soft tissues, a procedure using keratinized mucosa and connective tissue grafting was indicated that is already established in the literature [7].

**Figure 4:**
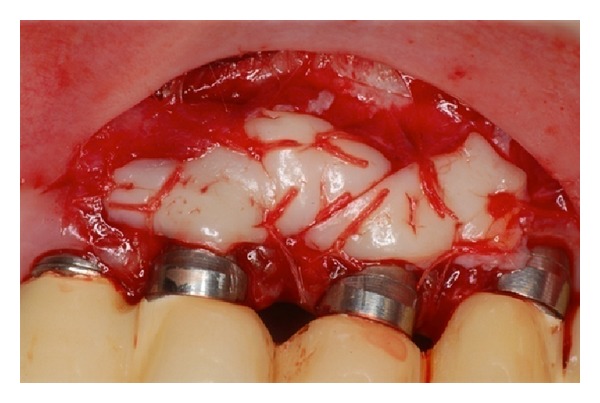
Graft sutured into the recipient site only in keratinized mucosa and periosteum.

The surgical procedure was carried out following the classical technique with the region of the tuberosity as the donor area, which had a surplus of sufficient tissue to perform this procedure. After complete stabilization of the graft on the receptor site, keratinized mucosa and adjacent periosteum (Figure 4), the patient was released and was prescribed a nonsteroidal anti-inflammatory, every 12 hours for 3 days.

**Figure 5:**
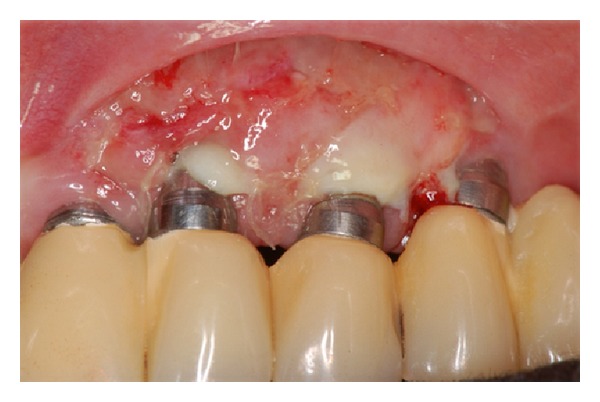
Postoperative aspect 7 days after surgery.

**Figure 6:**
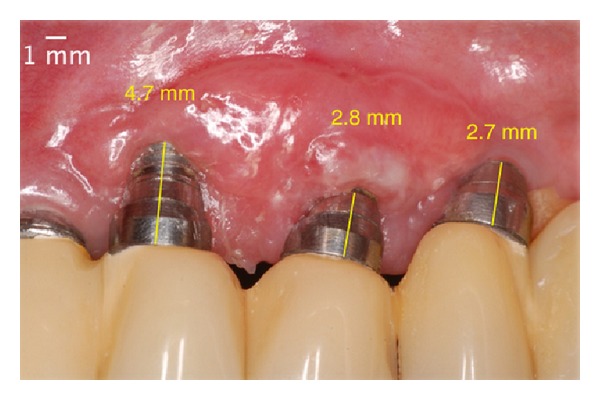
Postoperative aspect 21 days after surgery: the exposure of threads in the element 23 still exists.

**Figure 7:**
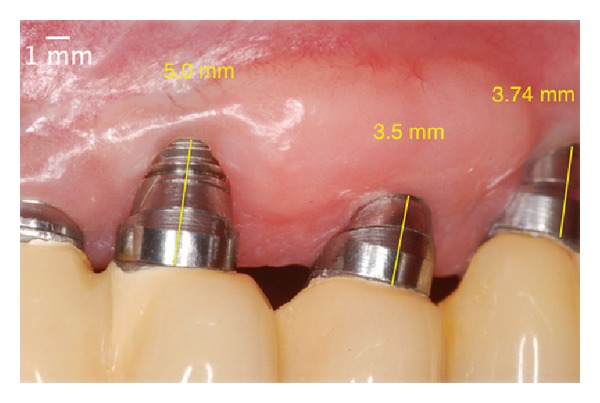
Postoperative aspect 90 days after surgery: indication for subepithelial connective tissue graft.

**Figure 8:**
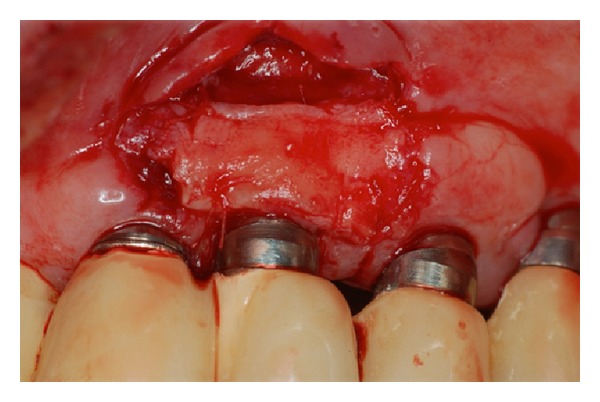
Connective tissue graft sutured to the recipient site.

**Figure 9:**
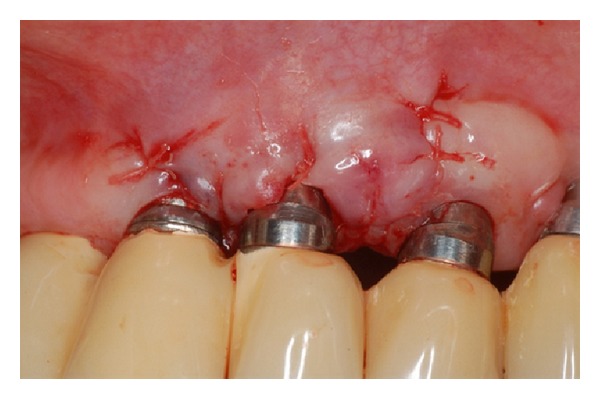
Immediate postoperative aspect after subepithelial connective tissue graft.

Seven, 21, and 90 days, postoperatively, the patient returned to the clinic with a satisfactory status regarding the success of the technique of grafting but without the covering of exposed threads (Figures 5, 6, and 7). Three months later, this patient underwent a new procedure for soft tissue graft, with the intention of covering the threads that were still exposed in the implant located in the area of tooth number 23. At this stage, only a connective tissue graft with a coronally advanced flap was planned. This procedure was performed following the classic technique described to cover exposed roots [11]. During this procedure, the donor site was the palatal region, extending from the distal of canine to mesial of the upper left first molar. In both of the surgical techniques, citric acid and tetracycline were used on the exposed surface of the implant (Figures 8 and 9).

**Figure 10:**
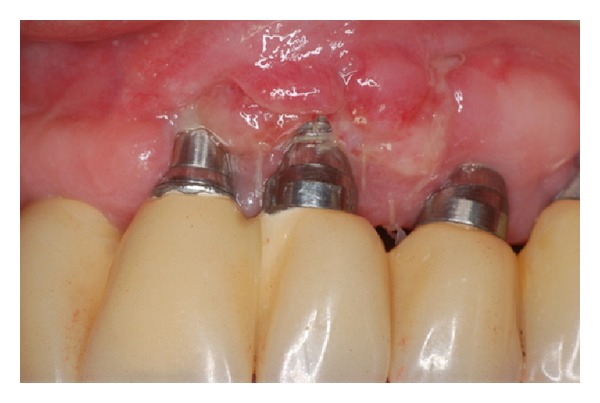
Postoperative aspect 7 days after surgery.

**Figure 11:**
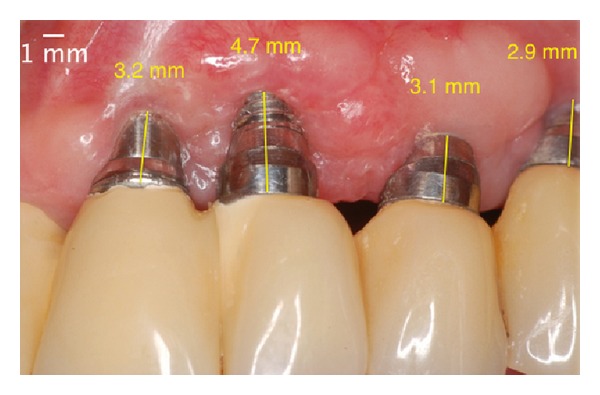
Postoperative aspect 21 days after surgery: note that the connective tissue graft failed to cover exposed threads.

**Figure 12:**
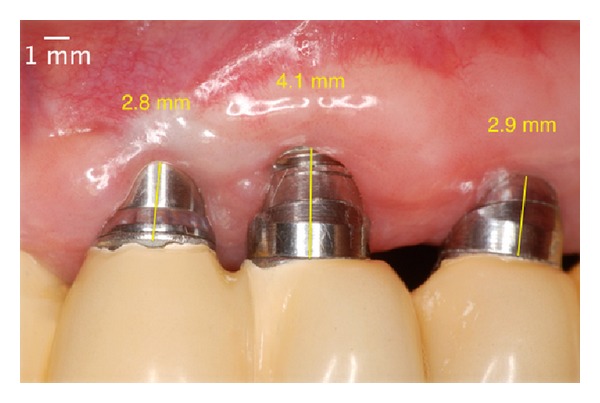
Postoperative aspect 90 days after the second surgery: presence of the phenomenon of “creeping attachment” with partial coverage of the implant's threads.

**Figure 13:**
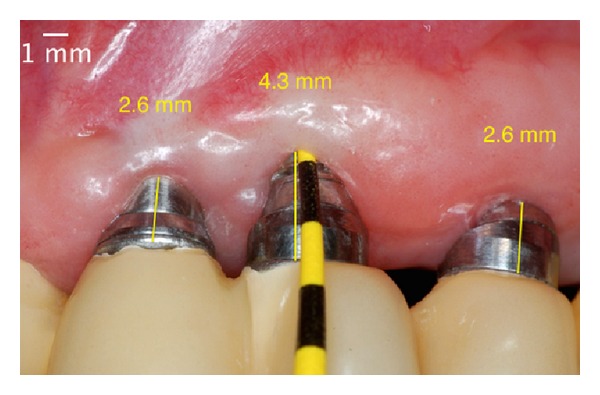
Postoperative absence of peri-implant pocket in 6 months.

**Figure 14:**
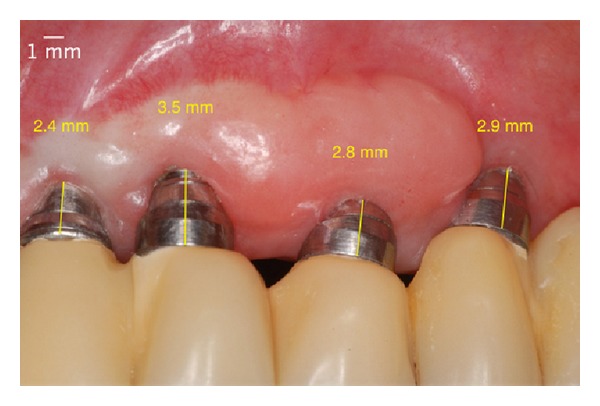
One year after the surgery. Note the almost total coverage of the implant platform.

**Figure 15:**
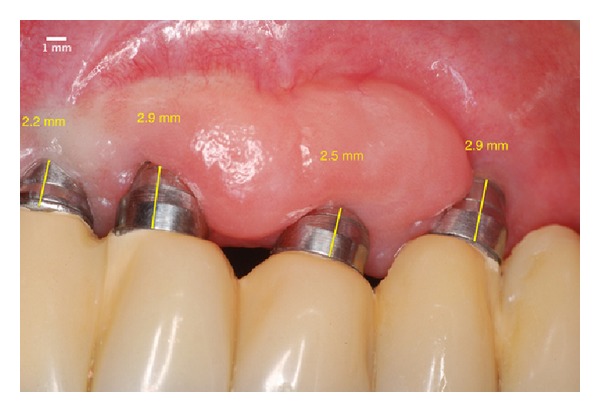
Two years after the surgery. Covering all of the threads of the implant and the platform due to the phenomenon of “creeping attachment.”

In the controls after 7 and 21 days, the latter procedure, despite creating a volume of soft tissue, failed to cover the threads of the implant (Figures 10 and 11). Because of this, this patient was kept on peri-implant maintenance therapy. Ninety days after the second surgery, there was coronal migration of peri-implant mucosa, which then covered 2 of the 3 exposed threads (Figure 12). Until then, this phenomenon, called “creeping attachment,” had only been described in roots. Periodical recall appointments were performed and a survey of peri-implant sulcus depths was completed noting the absence of pockets 6 months after the surgery (Figure 13). The cervical tissue migration still occurred after 1 year (Figure 14) until it stabilized during the postoperative period of 2 years (Figure 15), where we observed a complete coverage of the threads and the implant platform in the region of tooth number 23, leaving only the vestibular portion of the metallic collar inherent to the angled abutment.

### 2.2. Case 2

**Figure 16:**
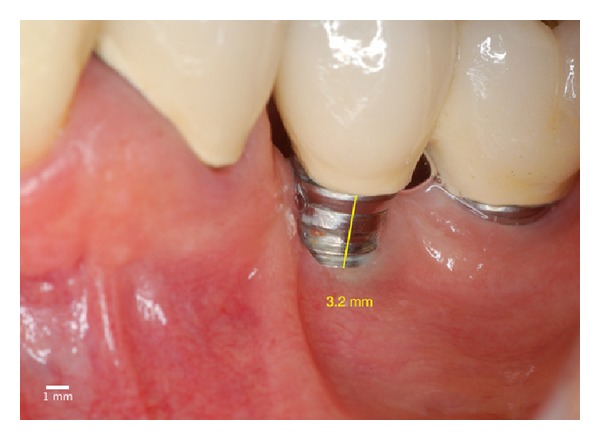
Initial appearance. Note exposure of threads and the absence of keratinized mucosa.

**Figure 17:**
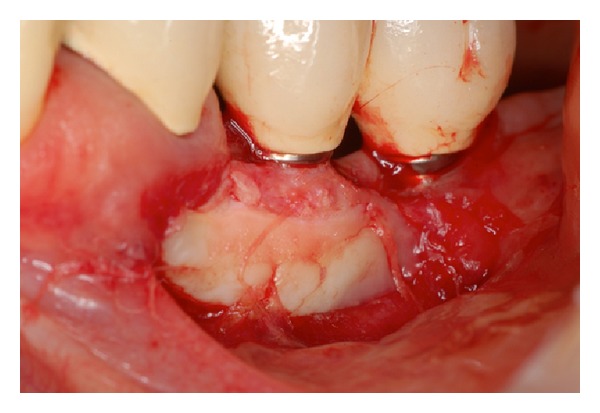
Graft sutured into the recipient site only in keratinized mucosa and periosteum.

**Figure 18:**
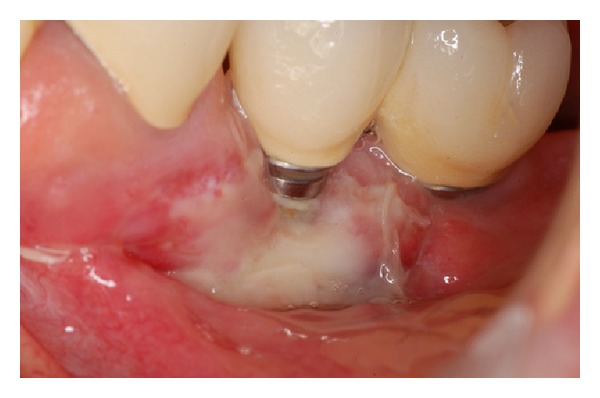
Postoperative aspect 7 days after surgery.

**Figure 19:**
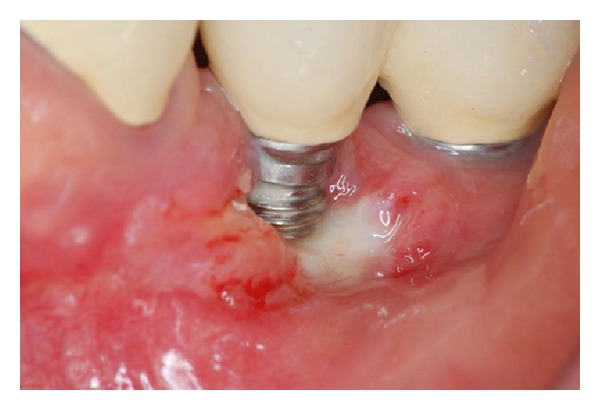
Postoperative aspect 14 days after surgery.

**Figure 20:**
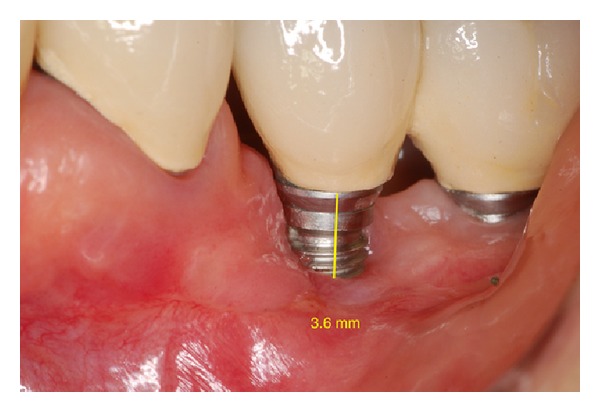
Postoperative aspect 21 days after surgery: note that the masticatory mucosal tissue graft failed to cover exposed threads but gained soft tissue volume.

**Figure 21:**
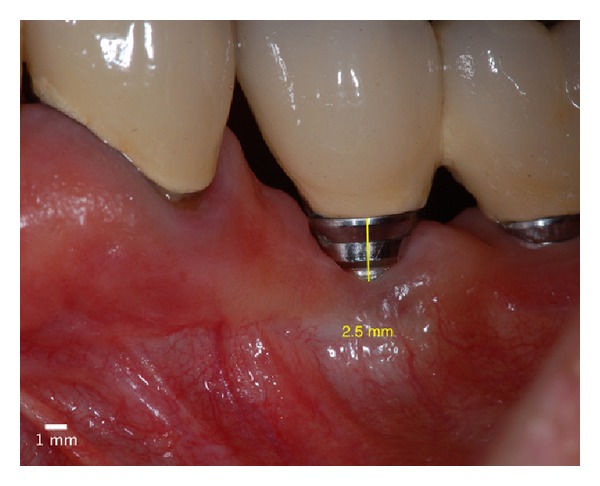
Two years after the surgery. Covering almost all of the threads of the implant due to the phenomenon of “creeping attachment”.

The patient reported difficulty in brushing due to painful stimuli. Clinical examination showed absence of keratinized tissue on the buccal surface of the implant in the area of tooth number 35 (Figure 16). To solve this problem, the technique of free gingival keratinized and connective tissue grafting was proposed, as previously described (Figure 17). After the postoperative controls of 7, 10, and 21 days, the graft did not achieve a satisfactory outcome for increasing the width of keratinized mucosa, but only slight augmentation of the soft tissue (Figures 18, 19, and 20) was noticed. A two-year follow-up postoperative visit showed the growth of coronary soft tissue, with almost total coverage of the threads of the implant, characterizing the phenomenon of “creeping attachment” (Figure 21).

## 3. Discussion

The literature contains few reports on “creeping attachment” and all have been reported surrounding teeth [12–16]. The vast majority of these were reported in areas of the mandibular incisors and only one was reported in a maxillary posterior tooth restored with a metal crown. The literature shows no case reports of “creeping attachment” around implants. When we used the key words to search in PubMed: creeping attachment dental implants, only one article was related [17] to our research, which showed how rare this phenomenon is around implants. It is known that the soft tissue behaves differently on implant surfaces. Histological studies show a parallel behavior of fibers on the surface of the implant or prosthetic abutment [18]. Several studies have been proposed to test different surfaces of the implant that would stimulate the straight insertion of the soft tissue fibers of which only one was able to be proven convincingly [19].

Such characteristics make the presence of the growth of coronary soft tissue more difficult. This paper presents two clinical cases in which soft tissue surgeries were used to stabilize a process of peri-implant disease and increase the quality of peri-implant mucosa. After a postoperative follow-up of 2 years, the phenomenon of “creeping attachment” was noted in which threads were covered with machined implants placed slightly in the buccal direction. Although the literature does not present consensus on the need for keratinized mucosa around implants [2], this group believes that the presence of this tissue allows greater comfort for hygiene by the patient and higher mechanical strength of the mucosa for brushing forces as well as providing marginal tissue homeostasis.

It was previously reported that root etching enhances the coronal growth [20–22]. In these cases, a chemical treatment of exposed threads was performed, where the graft would lie. Unlike teeth, this procedure relates only to the chemical decontamination of the thread, where the graft would rest, as implant surfaces have a high degree of difficulty of decontamination, where mechanical disinfection can be accomplished only with Teflon curettes. A series of studies confirms this type of procedure during surgery involving surfaces contaminated due to oral exposure or by peri-implant disease [22]. Various substances have been used for this purpose including citric acid (used in these cases) which showed good results [23].

This phenomenon of “creeping attachment” is present 1–12 months after surgery. However, as in other articles, an increase in peri-implant mucosa after that period was also observed. This amount of gingival and mucosal growth still not be expected and to achieve the inclusion of fibers in both roots and implant abutments, further clinical studies are needed. The peculiarity of this case is the presence of “creeping attachment” around implants surfaces and angled abutments, decreasing the exposure of the metal collar which is the principal limitation of using this kind of abutment.

Although the phenomenon of “creeping attachment” around implants was observed, it is not yet possible to quantify its growth. This paper shows that this coronal migration is possible over decontaminated surfaces.
